# Heart rate variability is enhanced during mindfulness practice: A randomized controlled trial involving a 10-day online-based mindfulness intervention

**DOI:** 10.1371/journal.pone.0243488

**Published:** 2020-12-17

**Authors:** Ulrich Kirk, Johanne L. Axelsen

**Affiliations:** Department of Psychology, University of Southern Denmark, Odense, Denmark; Scuola Superiore Sant'Anna (SSSA), Pisa and Fondazione Toscana G. Monasterio (FTGM), Pisa, ITALY

## Abstract

**Objectives:**

The goal of the present study was to probe the effects of mindfulness practice in a *naturalistic* setting as opposed to a *lab-based* environment in the presence of continuous heart rate variability (HRV) measurements. The specific experimental goals were to examine the effects of a brief 10-day online-based mindfulness intervention on both *chronic* and *acute* HRV responses.

**Method:**

We conducted a fully randomized 10-day longitudinal trial of mindfulness practice, explicitly controlling for practice effects with an active-control group (music listening) and a non-intervention control group. To assess c*hronic* cardiovascular effects, we asked participants in the 3 groups to complete 2-day HRV pre- and post-intervention measurement sessions. Using this experimental setup enabled us to address training effects arising from mindfulness practice to assess physiological impact on daytime as well as nighttime (i.e. assessing sleep quality) on the underlying HRV response. To assess *acute* cardiovascular effects, we measured HRV in the 2 active intervention groups during each of the 10 daily mindfulness or music sessions. This allowed us to track the development of purported training effects arising from mindfulness practice relative to the active-control intervention in terms of changes in the HRV slope over the 10-day time-course.

**Results:**

Firstly, for the acute phase we found increased HRV during the daily practice sessions in both the mindfulness and active-control group indicating that both interventions were effective in decreasing acute physiological stress. Secondly, for the chronic phase we found increased HRV in both the day- and nighttime indicating increased sleep quality, specifically in the mindfulness group.

**Conclusion:**

These results suggest causal effects in both chronic and acute phases of mindfulness practice in formerly naïve subjects and provides support for the argument that brief online-based mindfulness interventions exert positive impact on HRV.

## Introduction

Mindfulness practice has been framed as a technique that may promote well-being, which to some extend has been scientifically demonstrated through studies showing reduced self-reported stress (e.g. [1–3]) and improved self-reported sleep quality [4–8]. However, mindfulness has increasingly come under scrutiny in terms of difficulties with defining mindfulness, and for lacking important methodological issues for interpreting results from investigations of mindfulness and its purported effects [9]. In line with this criticism, the majority of studies on the issue of stress reduction and sleep quality have assessed mindfulness using self-report measures [10]. However, in the nascent field of wearable technology there are purported ‘objective’ physiological tools available to measure stress and sleep quality [9, 11, 12]. For example, heart rate variability (HRV) provides a powerful tool for observing the interplay between the sympathetic and parasympathetic nervous system [13–15].

There have been some, but limited, research (for reviews see [16, 17]) showing that mindfulness exerts beneficial effects on the cardiovascular system [18–28]. The majority of these studies have focused on acute changes from being in a ‘mindful state’, while some studies cited above have investigated changes in resting baseline HRV between long-term mindfulness practitioners and novices (i.e. chronic changes).

Investigations into the immediate physiological effects of mindfulness practice have revealed increased activity in HRV [22, 29]. Also, long-term mindfulness retreats have been shown to increase HRV [21, 24]. The interpretations of such increases in HRV and dominance of the parasympathetic nervous system (PNS) during mindfulness may partly be caused by changes in respiration which is modulated by the vagus nerve [14, 30, 31], and that respiration via awareness of breathing is central to mindfulness practice [32]. Indeed, studies have demonstrated that respiration rate is decreased and the HRV response increased during mindfulness [18, 33, 34]. Thus, it may be that respiratory rate should be considered as a metric reflecting decreased sympathetic drive during formal mindfulness practice. This raises an open question: Is the respiratory component only present during formal mindfulness practice (i.e. an *acute state-dependent effect*) or is it a *trait-dependent effect* emerging in the course of practicing mindfulness over time (i.e. a *chronic effect*)?

### The present study

The overall goal of the present study was to investigate the purported effects of mindfulness in a *naturalistic* setting as opposed to a *lab-based* environment through the lens of HRV, while at the same time examine the distinction between *acute* and more *chronic* HRV changes arising from mindfulness practice. To meet these experimental goals, we designed a study that tried to bifurcate acute and chronic effects of mindfulness practice. We employed a fully randomized 10-day online-based longitudinal mindfulness intervention, whereby we controlled for practice effects with an active-control group as well as a non-intervention control group in the context of continuous HRV measurement. In that cross-sectional studies cannot demonstrate causality, and wait-list designs are confounded by unmatched practice-effects and efforts [35], we decided to employ a longitudinal design involving music listening as an active-control intervention with similar practice duration and demand characteristics as the mindfulness group (see Materials and Methods for a description of the two active interventions). As previous studies in mindfulness have shown increased attentional control arising from mindfulness practice [36–39] presumably through interoceptive nonjudgmental awareness [35, 40–42], we chose music as an active-control intervention which we expected would deemphasize these elements, thereby isolating the components of action in mindfulness practice.

### Employing HRV to track cardiovascular effects of stress

It is generally assumed that HRV is a measure of beat-to-beat variability in heart rate (HR) that is mediated by the autonomic nervous system (ANS). The sympathetic nervous system (SNS) increases the heart’s contraction rate and force (cardiac output) and decreases HRV, which is needed during exercise and mentally or physically stressful situations. Conversely, the PNS slows the heart rate and increases HRV to restore homeostasis. This natural interplay between these two systems allow the heart to quickly respond to different situations and needs based on the context [43]. The root mean square of successive differences between normal heartbeats (RMSSD) is considered to represent the beat-to-beat variance in heart rate (HR) and is the primary time-domain measure used to compute the vagally mediated changes reflected in HRV [14]. The primary frequency-domain measure is the high frequency HRV (HF-HRV) component (0.15 to 0.40 Hz) which estimates inhibitory vagally induced PNS input and LF/HF ratios [14]. We report both results from time-domain measures (RMSSD) and frequency-domain measures (HF-HRV and LF/HF ratios) whilst also summarizing additional time- and frequency domain measures (see S1 and S2 Tables).

### Chronic cardiovascular effects of mindfulness practice

To assess chronic effects of mindfulness practice on the underlying HRV response, we asked participants in the 3 groups to initially complete 2-days (48 hours) of continuous HRV measurement, which constituted a pre-intervention chronic phase (see Material & Methods for a description of the definitions of *chronic* and *acute phase* measurements). A similar procedure was implemented after completion of the 10-day interventions, that is participants belonging to the 3 groups were asked to discontinue the practice during the 48-hour HRV measurement session, which constituted a post-intervention chronic phase. This setup allowed us to probe chronic effects of mindfulness practice on the HRV response in that no formal mindfulness practice took place neither in the pre or the post intervention sessions. We expected the active control group would not show an effect on respiration rate that may be a metric reflecting decreased sympathetic drive during formal mindfulness practice as mentioned above, and therefore may not have an impact on chronic HRV effects in the active control intervention or in the non-intervention control group. This aspect of the experimental design enabled us to isolate the components of action in mindfulness practice. In addition, based on findings showing that mindfulness reduces self-perceived stress [1–3], our first hypothesis (H1) was that mindfulness practice would increase the HRV response during daytime in the mindfulness group post-training compared to pre-training and across groups.

### Acute cardiovascular effects of mindfulness practice

To assess acute effects of mindfulness practice on the underlying HRV response, we measured HRV in the two active intervention groups during each of the 10 daily mindfulness or music sessions, which constituted daily acute phases of HRV across the intervention period. This allowed us to track the development of mindfulness skills relative to the music group in terms of changes in the HRV slope over the 10-day time course. The instructions for the practice of mindfulness involves intentionally directing attention to one’s experience in the present moment [44]. This practice entails frequently becoming distracted and returning the attention to the present moment, by centering awareness on present moment experiences and thereby enhancing attentional capacity. Novice practitioners often experience that mindfulness practice entail frequent distractions and that intentional focus has wandered [45]. Given that the participants were novices to the practice of mindfulness, we expected that this should be reflected as practice effects in the mindfulness group. Specifically, as mindfulness practice over the 10-day time course would reflect a practice effect and thereby increase the HRV response, our second hypothesis (H2) was that the HRV response in the mindfulness group would be significantly elevated over the 10-day practice period. Furthermore, based on previous findings showing that respiration rate is decreased and the HRV response increased during mindfulness even without instructions to alter (i.e. slow) breathing [18, 33, 34], our third hypothesis (H3) was that mindfulness practice would decrease respiration rate in the acute practice phase and not in the chronic phase. Attenuated respiration rate or longer exhalations relative to inhalations, often seen in mindfulness practice [21, 22, 24, 29], exert immediate physiological effects caused by parasympathetic activation, such as decreased oxygen consumption, decreased heart rate and blood pressure, and increased HRV [46]. As such, we sought in H3 to address whether slowed respiration would be present exclusively during formal mindfulness practice (i.e. acute phase) or whether reduced respiration was also present outside of formal mindfulness sessions (i.e. chronic phase).

### Cardiovascular effects of sleep as a function of mindfulness practice

Our experimental setup furthermore enabled us to address the effects of mindfulness on sleep quality. Sleep is a fundamental part of life, and serves as a biological investment associated with growth, repair, and maintenance of bodily functions [47]. Poor sleep is associated with increased risk of cardiovascular disease [48] and associated with mood and anxiety symptomatology [49]. As sleep exerts an effect on HRV [50], studies have associated poor sleep quality with elevated sympathetic activity and suppressed parasympathetic activity [51].

Based on numerous findings showing that mindfulness exerts a positive effect on self-perceived sleep quality [4–8], our fourth hypothesis (H4) was that mindfulness practice would increase the HRV response during sleep in the mindfulness group post-training compared to pre-training and across groups.

Finally, we collected self-report data from the Perceived Stress Scale (PSS) [52], the Mindfulness Attention Awareness Scale (MAAS) [53] and the D3 Sleep Quality Index (D3SQI) to access differences across groups. In addition we analyzed home practice adherence data explicitly controlling for practice effects with an active-control group to probe if mindfulness practice dose-response impacted the HRV response.

## Materials & methods

### Participants

A total of 99 healthy volunteers participated in the study. 9 participants either dropped out or exhibited >10% missing data in the HRV-pre/post measurements (3 participants in the mindfulness group; 4 participants in the music group; 2 participants in the control group). Thus, the total number of participants from which data could be collected was 30 in the mindfulness group, 30 in the music group and 30 in the control group. Age and gender distributions are listed in Table 1.

**Table 1:** Behavioral data for the three groups, shown as mean and standard deviation collected pre and post-intervention.

	Mindfulness group	Music group	Control group
N (females)	30 (21)	30 (20)	30 (22)
Age (years)	36.8 ± 12.74	36.3 ± 9.18	36.6 ± 9.51
Practice dose-response (min)*	225.9 ± 31.2	233.5 ± 31.6	-
MAAS pre	3.1 ± .6	3.1 ± 1.0	3.1 ± 1.0
MAAS *post*	3.9 ± .7	3.2 ± .9	3.1 ± 1.1
PSS pre	19.1 ± 6.6	19.5 ± 5.7	18.2 ± 4.7
PSS *post*	14.1 ± 5.6	18.0 ± 5.6	17.9 ± 6.5
D3SQI pre	25.2 ± 3.8	24.0 ± 3.9	25.1 ± 4.0
D3SQI *Post*	28.4 ± 5.6	24.6 ± 4.9	25.2 ± 4.6

*The amount of practice was calculated as the total minutes of practice during the 10-day interventions.

Abbreviations: MAAS (Mindfulness Attention Awareness Scale); PSS (Perceived Stress Scale); D3SQI (D3 Sleep Quality Index).

### Recruitment

Recruitment for the current study involved online-based advertisement campaigns through the University of Southern Denmark’s Facebook-page. The study was framed as a stress reduction study. Recruitment information furthermore informed that that the study involved either a mindfulness, music or a non-intervention control group lasting 10 days with a required 20–30 min. of daily training using an app-based platform (either mindfulness or music). In addition, recruitment information included that participants would be assigned to one of the three groups in a random manner, which eliminated any self-selection bias across the groups. Participants were informed that they in addition to one of the two intervention *training-apps* (either mindfulness or music) would be required to complete questionnaires during the intervention period. The next stage of the recruitment process involved that interested participants were provided with written information specifying the study’s logistics and requirements. After having agreed to the study requirements in writing, participants were invited to a meeting in which each participant individually received verbal information about the physiological recording procedure and information about when to fill in the questionnaires. This information included that participants at any time during the study had the option to discontinue their participation in the study. Participants were informed that the app-based platforms (i.e. the mindfulness and music interventions) utilized in the study ran on both Android and IOS, and thus required that participants had access to a smartphone for the study duration. After this information was provided to participants, they were given an option to ask questions about the study before being asked to sign the consent form. Following consenting to the study, participants were informed and were visually shown the physiological recording equipment and briefed regarding the experimental procedures. They received this information both verbally and in writing (handouts). Exclusion criteria were previous experience with mindfulness meditation, and current psychiatric illness or psychiatric medication intake or not owning a smartphone. Inclusion criteria required that all participants were between 21 and 60 years of age, and interest in receiving a free stress reduction intervention. Participants received monetary compensation for their participation in the study corresponding to DKK 400 (approximately USD 60). All procedures were conducted in accordance with the local ethical committee (Videnskabsetisk Komité for Region Syddanmark–Ethics approval ID S-20170199).

#### Experimental procedures

The randomization sequence was determined after study recruitment but before study launch. Specifically, participants were allocated to either the mindfulness, active-control music or non-intervention control group in a random manner. The 99 participants who volunteered for the study during the recruitment period (which took place from November to December 2019) were randomized into one of the 3 groups. This randomization procedure ensured that the data collection period (which took place from January 2020 to August 2020) was spread out across the 3 groups. Participants were not informed about group allocation until arrival to the lab for HRV measurement. Sequence generation and randomization was performed by the research team, who were not formally blinded to group allocation.

Participants were informed in the lab regarding the procedures related to the 10-day intervention. Participants were given instructions about the 2-day/48 hours continuous HRV measurement that would take place prior to initiating the active intervention (mindfulness or music listening). Prior to the HRV measurement, participants were informed to refrain from alcohol and nicotine in order to avoid known influences of these factors on autonomic activity [31, 54, 55]. Participants were instructed to not to engage in intense physical activity for the 48-hour period but were otherwise asked to maintain their daily and nightly routines. Both the pre and post measurement periods were conducted on weekdays. Following the 48-hour pre-measurement, the participants were instructed to initiate the 10-day mindfulness or music intervention. During the daily mindfulness or music sessions in the course of the 10-day intervention, participants’ HRV response were recorded. Upon completion of the 10-day intervention, participants completed another 48-hour HRV measurement. The resulting time course containing pre and post measurements and data from practice sessions for each participant was extracted from the HRV-monitor upon completion of the study and was processed for further analysis (see *Physiological measures* below).

Furthermore, during the visit to the lab, participants in the three groups were provided with oral and written instructions for usage of the HRV-monitor that was employed in the study. Having received practice and demonstration of montage of the electrodes and HRV-monitor, participants in the two active interventions (i.e. mindfulness and music) were instructed in how to complete the daily practice session at home. Specifically the instructions included that during the daily sessions (mindfulness or music listening) participants were asked to sit in an upright position on a chair or on a cushion quietly by themselves and follow the guided mindfulness session (i.e. mindfulness group) or listen to the music (i.e. music group) for the entire duration of the session. All participants subjectively recorded home practice using a paper logs that they were provided with by the research team (see *Compliance data* below). Participants were instructed to initiate HRV recording 5–10 min prior to initiating the daily sessions to allow for calibration, and furthermore asked to complete the daily practice sessions at approximately the same time (between 8am-6pm) and not to engage in intense physical activity approximately 2–3 hours before the session.

The *acute cardiovascular effects* were defined and operationalized for the purpose of this study as HRV measurement phases during which participants formally practiced mindfulness or were listening to music. HRV was captured and time-locked using the cross-checked timestamps derived from the training apps (see *Compliance data* and *Interventions*: *Mindfulness and music* below). This entailed a dataset of 10 consecutive daily time courses with a duration of 20 min for the initial 5 days and 30 min for the last 5 days for each participant where they practiced either mindfulness or were listening to music.

By contrast the *chronic cardiovascular effects* were defined and operationalized as HRV measurement phases conducted either at baseline (i.e. pre) or following (i.e. post) the 10-day intervention. Importantly, participants were instructed not to practice mindfulness or listen to music for the duration of these measurement periods. This entailed a continuous 48-hour measurement phase for each participant both pre and post intervention. Note that the 48-measurement phases were binned in segments according to the diurnal rhythm (see *Physiological measures* below). These measurement phases were initiated immediately before the intervention and the following day after completion of the intervention.

### Interventions: Mindfulness and music

#### Mindfulness intervention

The mindfulness intervention consisted of a 10-day app-based program provided by Headspace (https://www.headspace.com/). Participants did not receive an introductory session to the mindfulness or music programs but were provided with written instructions related to installation of the training app and usage for the 10-day intervention. The content of the training was based on well-established concepts and practices within the mindfulness literature [32] and entailed daily practice in guided mindfulness meditation, with instructions delivered through short animated videos and sound files in the app. The training program centered on mindfulness meditation, which included focusing on a selected object (i.e. the body or the breath), monitoring the activity of the mind, noticing mind-wandering, and developing a non-judgmental orientation toward one’ s experience (i.e., equanimity).

The mindfulness group was instructed to follow an introductory course to mindfulness in the Headspace app with two levels, namely ‘Basics I-II’. The program entailed that participants completed ‘Basics I’ for the initial 5 days with a daily duration of 20 min, and the ‘Basics II’ program for the remaining 5 days with a daily duration of 30 min. The Headspace app has been applied in previous research demonstrating effects pertaining to stress-relief such as overt self-reported stress [56], self-reported well-being [57, 58] and self-reported mindfulness [59, 60].

By examining user data provided by the app developers on how much time each subject had spent meditating with the app, we could confirm that all participants showed acceptable adherence to the program (>80%). Participants were informed of this and consented to us gaining access to their user data before entering the study.

#### Music intervention

We employed an active-control condition (listening to music), which we also made available using an app-based platform to structurally match the active-control intervention on content not specific to mindfulness, while in addition controlling for nonspecific treatment effects such as placebo, social support, and demand characteristics [61, 62].

The music used in the study was instrumental music and there were in total 60 music compositions. The music was organized according to different playlists in the app, specifically ‘focus’, ‘binaural beats’, and ‘piano’. Each of the 3 playlists consisted of 20 tracks with a duration of between 2 to 4 min. Participants were instructed to freely select which playlists to listen to and they were free to listen to any or all 3 playlists during the study. The daily listening requirement was 20 min for the initial 5 days, and 30 min for the remaining 5 days for the music group to match and allow balanced comparison across the mindfulness intervention group.

By examining user data provided by the app developers on how much time each subject had spent listening to the music available in the app, we could confirm that all participants showed acceptable adherence to the program (>80%). Participants were informed of this and consented to us gaining access to their user data before entering the study.

#### Non-intervention control

The non-intervention control group were asked to maintain their daily and nightly routines for the 10-day period between the pre and post 48-hour HRV measurement period and were explicitly asked not to perform mindfulness or listen to music during this period. Acute physiological data was not collected from the non-intervention group in that there was no uniform activity level (as opposed to the two active intervention groups) that this group was asked to perform.

#### Compliance data

Participants in both app-based intervention groups were instructed to follow the programs in full to receive the maximum benefit of the interventions and complete the daily training/listening requirements at any time during the day that fitted with their schedule from 8am– 6pm.

Participants were provided with a log in which they were asked to fill in the time during the day when they completed the daily practice. It was emphasized that self-reports should accurately reflect their practice so as to discourage dishonest reporting. The log was handed over to the experimenters upon completion of the interventions.

Both apps (i.e. mindfulness and music) contained a function that tracked the timestamps during which time the participants used the app. This usage information was available to participants to keep track of their daily usage during the study. In addition, the time course containing each completed practice session for each participant was extracted from the apps upon completion of the study by the experimenters and was processed for further analysis. Specifically, the usage data generated from the app was cross-checked with the self-report logs for each participant. The physiological data was adjusted and time-locked with the onset timestamp provided in the apps. We included data in which participants completed >80% of a practice session. The mean practice data is reported in the Results section (Table 1).

### Psychological measures

3 questionnaires were employed pre and post the 10 days intervention using an app-based platform (https://www.datacubed.com/). The pre-questionnaires were filled in by participants prior to initiating the 48-hour HRV pre-measurement, while the post-questionnaires were filled in after completion of the 48-hour HRV post-measurement. However due to an error in the app (https://www.datacubed.com/), datapoints from 13 participants (3 in the mindfulness group; 4 in the music group; 6 in the control group) were not captured and were thus lost.

Initially, all participants were asked to complete the PSS [52]. The PSS is a 10-item scale designed to measure the perception of stress. Furthermore, all participants were asked to complete the MAAS [53]. The MAAS is a 15-item scale designed to assess dispositional mindfulness. Finally, participants were asked to complete the D3SQI, which is a 34-item questionnaire. The gold standard for assessment of sleep quality is polysomnography [63], however the Pittsburgh Sleep Quality Index (PSQI) [64] has been demonstrated to have cardiovascular prognostic value [63], and as the D3SQI has been constructed to parallel the PSQI, the D3SQI was thus chosen to be applied in the current study to assess sleep quality.

The mean data from the participants psychological measures for the three intervention groups are reported in Table 1.

### Physiological measures

#### Physiological acquisition

HR was recorded as beat-to-beat intervals with the Firstbeat Bodyguard II HRV monitor (Firstbeat Technologies Ltd., Jyväskylä, Finland) that have been previously applied in research and validated with standard physiological monitoring systems used in clinical and laboratory settings [65–67]. Bodyguard 2 is a wearable lightweight monitor attached on the chest using two ECG electrodes (Ambu Ltd., Ballerup, Denmark) for measuring 24h HRV (RR-intervals) including respiratory measures.

#### Physiological signal processing

The HRV measurements conducted in this study were performed according to the guidelines of the Task Force of the European Society of Cardiology and the North American Society of Pacing and Electrophysiology [31]. HRV allows to quantify the change in the time intervals between consecutive heart beats and refer to an index of SNS activity and PNS activity at any given time [13]. Quantification of HRV parameters can broadly be classified into time and frequency domain measures. The primary time-domain measure is RMSSD and reflects the beat-to-beat variance in heart rate (HR). RMSSD is typically used to estimate vagally mediated changes reflected in HRV [14]. RMSSD is reported in milliseconds (ms). The primary frequency-domain measure is high frequency HRV (HF-HRV) component (0.15 to 0.40 Hz) which estimates inhibitory vagally induced PNS input and LF/HF ratios. In following these standardized procedures, we report both RMSSD, HF-HRV and LF/HF ratios in this study. Furthermore, to gain a comprehensive insight of the ANS adaptation to the mindfulness practice employed in the current study, we also report other measures in the temporal and frequency domain (see Supporting Information).

All raw physiological data was processed for time- and frequency-domain parameters using the Kubios analysis software (version 3.4). The recorded data was imported to Kubios to calculate R-R intervals and associated variability [68]. Examination of the electrocardiogram data (ECG) ensured that the autonomic R-wave detection algorithm had been performed satisfactorily. Artifact removal for the HRV was performed manually using the artifact correction tool to detect R-R intervals provided by the Kubios software. When correction was applied, detected artifact beats were replaced using cubic spline interpolation. Spectrum analysis was computed using the Fast Fourier Transformation procedure provided by the Kubios software. Because of the skewed distribution the HRV variables were log transformed prior to exposing the data to statistical analysis. The HRV data was recorded continuously at the pre and post time-points for the 48-hour pre-measurement and 48-hour post-intervention measurement. The time course was broken up into 24-hour segments and calculated as daytime (16 hours) and nighttime (8 hours) means on a participant-by-participant basis. The data was segmented according to estimated sleep (8 hours) and wake hours (16 hours) across participants. Due to these extensive time courses, the HRV activity reported in this study is to be considered a combination of SNS activity and PNS activity at any given time [13].

### Statistical analysis

All data is presented in mean ± SD unless otherwise stated. The data from the chronic phase (i.e. pre-post) were analyzed separately from the acute data (i.e. each of the 10 daily mindfulness or music sessions). Assumptions of normal distribution and sphericity of data were checked accordingly. Greenhouse-Geisser correction to the degrees of freedom was applied when violations to sphericity were present. Mixed 2 × 3 ANOVAs were used to assess if there were differences pre and post intervention on the groups’ mean RMSSD, HS-HRV and LF/HF ratios during day or night and their respiration rate during day or night. Significant interaction effects from the mixed ANOVA were followed up with *t* tests. For the acute data a mixed 10 × 2 ANOVA were used to assess if the two active interventions had an acute effect on the groups’ RMSSD, HS-HRV, LF/HF ratios and respiration rate during the 10 intervention days. Significance was set at 0.05 (2-tailed) for all analyses. Pearson correlation analysis was conducted to investigate practice dose-response and change in the mindfulness and music groups’ RMSSD from pre to post measurement. Pearson correlations (R) were considered small = 0.1, medium = .24 and large = .37 as suggested by Cohen [69]. The effect sizes for the mixed measures ANOVAs were calculated as partial eta squared (*η*^*2*^*p*), using small = 0.02, medium = 0.13 and large = 0.26 interpretation for effect size [70]. The effect sizes for the *t* tests were calculated as Cohen’s d using small = 0.2, moderate = 0.5 and large effect 0.8 also suggested by [69]. All data analysis was conducted using the statistical packages for social science (SPSS version 26).

## Results

### Demographical and behavioral effects

Table 1 display descriptive statistics with means and standard deviations for the three groups. A one-way ANOVA was conducted to investigate possible differences between the group’s descriptive statistics. There was no significant age difference between the groups (F(2,87) = .022, *p* = .97). Likewise, there was no significant difference in the music and mindfulness groups’ practice dose-response (paired *t* = -1.50, df = 51, *p* = .14).

For the questionnaire data, at pre-intervention there were no significant differences between the three groups. A mixed ANOVA was used to inspect time (pre and post measurement) by condition (mindfulness, music and control) for the groups’ scores on MAAS, PSS and D3SQI questionnaires. For the MAAS there was a significant interaction between group and time (F(2,74) = 6.24; *p* = .003, *η*^*2*^*p* = .14). Follow up paired *t* test revealed that in the music (*p* = .73) and control group (*p =* .96) there were no significant changes in MAAS-score from pre to post measurement. However, in the mindfulness group there was a significant increase in MAAS-score from pre to post measurement (paired *t* = -3.9, df = 26, *p* = .001) indicating that the group’s subjective mindfulness level increased. For the PSS-questionnaire there was a significant interaction of time and condition (F(2,74) = 3.54: *p* = .034, *η*^*2*^*p* = .08). Follow up paired *t* tests revealed that both in the music (*p* = .30) and control (*p* = .85) groups there were no significant changes in PSS-score from pre to post measurement. However, in the mindfulness group there was a significant decrease in the PSS-score from pre to post measurement (paired *t* = 7.46, df = 26, *p* < 0.01) indicating significantly lower perceived stress for the mindfulness group. The questionnaire data on the D3SQI displayed no significant interaction between group and time (*p* = .53) There was a significant effect of time (F(1,74) = 5.21; *p* = .025, *η*^*2*^*p* = .06). A follow up paired t-test showed that only the mindfulness group had a significant higher score on the D3SQI from baseline to post measurement (*paired t* = -3.267, *p* = .003, df = 26), this was not the case for the music or the control group. Furthermore, there was a significant effect of group (F(2,74) = 3.90; *p* = .024, *η*^*2*^*p* = .09) with the mindfulness group showing a significant higher score on the D3SQI on post measurement than the two other groups.

### Chronic cardiovascular effects

**Fig 1:**
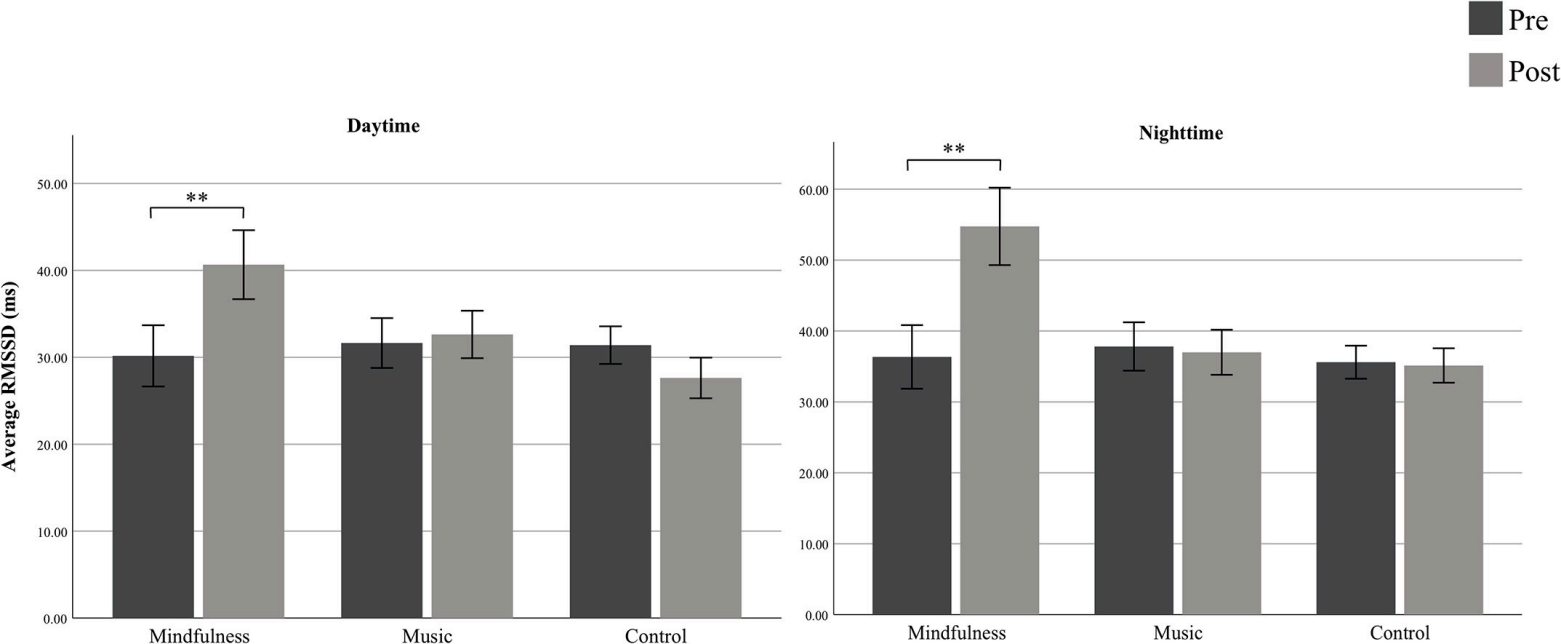
Group means of RMSSD (ms.) during daytime (left panel) and nighttime (right panel) for the chronic phase (i.e. pre and post measurement). The mindfulness-group showed at pre a mean RMSSD during daytime of 30.16ms. (SD = 12.2) and of 36.35ms. (SD = 15.6) during nighttime. At post-measurement the group showed a RMSSD mean of 40.65ms. (SD = 13.8) during daytime and 54.76ms. (SD = 19.0) during nighttime. The music group showed at pre a RMSSD-mean of 31.64ms. (SD = 10.7) during daytime and of 37.82ms. (SD = 12.7) during sleep. At post measurement the group showed a RMSSD-mean of 32.64ms. (SD = 10.2) during daytime and 37.0ms. (SD = 11.8) during nighttime. The control-group had a mean RMSSD at pre-measurement of 31.40ms. (SD = 8.2) during daytime and 35.61ms. (SD = 8.8) during nighttime. The control group showed a mean RMSSD at post measurement of 27.62ms. (SD = 8.8) at daytime and of 35.14ms. (SD = 9.2) during nighttime. Error bars are 95% CI.

To address H1 we computed the mean daytime RMSSD for the three groups (Fig 1, left panel). A mixed ANOVA was used to inspect time (pre and post) by condition (music group, mindfulness group and control group) for the groups’ RMSSD controlling for age and gender. There was a significant interaction of time and group condition (F(2,84) = 6.19; p = .003, η^2^p = .12). Follow up paired t-test showed that in the mindfulness group there was a significantly higher mean daytime RMSSD from pre to post measurement (paired *t* = -4.41, df = 48, *p* < .001). There were no significant differences in the active control group (*p* = .45) and the non-active control group exhibited a significant lower mean daytime RMSSD from pre to post (paired *t* = 2.79, df = 57, *p* = .007). We also computed the HF-HRV and the LF/HF ratio during daytime for the three groups controlling for age and gender. A mixed ANOVA did not reveal significant differences between group and time for HF-HRV (F(2,84) = 1.23, *p* = .37) or LF/HF ratio (F(2,84) = 1.42, *p* = .24).

To address H4, the mean nighttime RMSSD for the three groups was calculated (Fig 1, right panel). A mixed ANOVA was employed to inspect time (pre and post) by condition (music group, mindfulness group and control group) for the groups’ RMSSD during sleep with age and gender as covariates. There was a significant interaction of time and group condition (F(2,84) = 18.46; p < 0.01, *η*^*2*^*p* = .30). Follow up t-tests showed that in the music and control groups there were no significant changes in RMSSD during sleep from pre to post, however in the mindfulness group there was a significant increase in RMSSD during sleep from pre to post (paired *t* = -7.46, df = 48; p < 0.01). The HF-HRV and the LF/HF ratio during nighttime for the three groups controlling for age and gender did not reveal significant differences between group and time in a mixed ANOVA for HF-HRV (F(2,84) = 1.47, *p* = .22) or LF/HF ratio (F(2,84) = 1.82, *p* = .18).

### Acute cardiovascular effects

**Fig 2:**
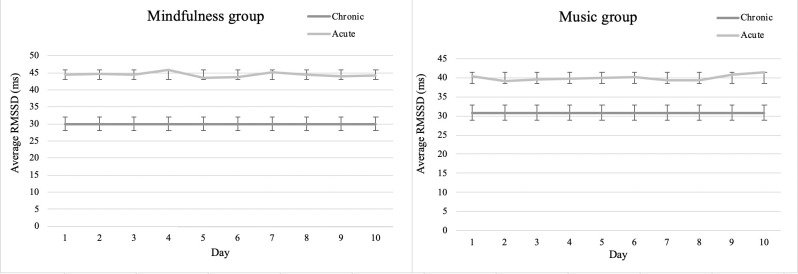
Group mean of acute RMMSD during the acute phase across the 10 intervention days. Visual representation of the mindfulness groups’ (left panel) and the music groups’ (right panel) acute RMSSD (*grey line*) on each of the 10 days of the music or mindfulness intervention as well as their pre-intervention daytime RMSSD shown as the mean from the chronic 48-hour pre-intervention phase (*black line*). Error bars are ±2 SD.

To investigate H2, namely music and mindfulness’ acute effect on heart rate variability, we investigated whether there was a difference in the groups’ pre RMSSD and their RMSSD while practicing mindfulness (Fig 2, left panel) or listening to music (Fig 2, right panel). Specifically, for the purpose of addressing H2, we used the daytime RMSSD from the chronic pre-measurement phase, i.e. the participants’ 48h HRV-measurement prior to the intervention, and in addition the acute RMSSD, i.e. from the 10 intervention sessions. Subsequently we computed a delta variable from participants’ acute RMSSD and subtracted it from the chronic daytime RMSSD. A mixed ANOVA controlling for age and gender, showed that there was no significant effect on the participants’ acute RMSSD between the two interventions. However, the mindfulness intervention produced a significant mean change in RMSSD of 12.99 ms (95% CI [8.42, 17.57]) and the music group produced a significant mean change in RMSSD 8.50 ms (95% CI [4.04, 12.97]) indicating an acute effect of both interventions. When looking at the HF-HRV and the LF/HF ratio during the acute phase for the two active intervention groups, we did not observe significant differences controlling for age and gender for either LF/HF ratio (F(9,29) = 1.44, p = .17) or HF-HRV (F(9,29) = 1.32, p = .24).

**Fig 3:**
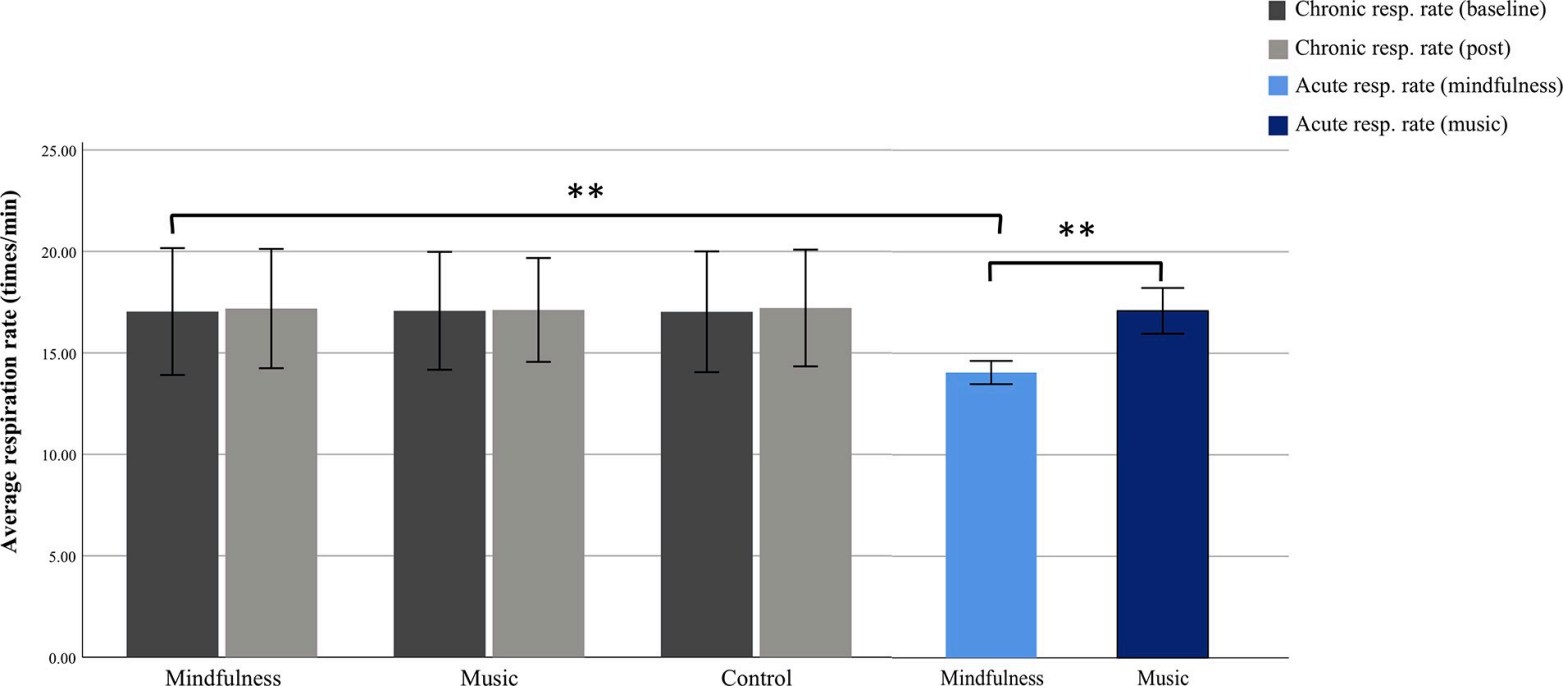
Group respiration rate for both the chronic and acute phase across groups. The mindfulness, music and control group’s mean respiration rate (times/min) measured during the chronic 48-hour pre- and post-intervention phase, and the mean respiration rate during the daily mindfulness or music sessions measured during the acute phase. The mindfulness group’s mean respiration rate was 17.10 times/min (SD = 1.67) at pre-intervention and 17.04 times/min (SD = 1.56) at post. The music group’s mean respiration rate was 17.18 times/min (SD = 1.31) at pre and 16.97 times/min (SD = 1.38) at post-intervention. For the control group the mean respiration rate at pre-intervention was 16.93 times/min (SD = 1.54) and 17.03 times/min (SD = 1.44) at post. Error bars are ±2 SD.

Furthermore, to address H3 we sought to investigate the effects of respiration rate in the two groups during the acute phase. The mean respiration rate for the mindfulness group during mindfulness practice across the 10 interventions days was 14.05 times/min (SD = .29), while the mean respiration rate for the music groups whilst listening to music was 17.09 times/min (SD = .5). The groups’ mean respiration rate from the chronic phase was calculated both pre and post intervention for the 48-hour period (Fig 3). The same procedure for the above-mentioned mixed ANOVA was used. That is, the participant’s baseline respiration rate, i.e. the 48-hour pre-intervention measurement from the chronic phase was subtracted from their acute respiration rate during either mindfulness practice or music-listening. There was a significant interaction effect of group (mindfulness vs. music) and time (10 intervention days) (F(9,29) = 3.52, p = .005, *η*^*2*^*p* = .52) when controlling for age and gender. Mindfulness practice produced a significant mean change on the participants’ respiration rate (-3.5 times/min [CI: -4.00, -2.68]), there were no such significant effect on participants’ respiration rate in the music group (Fig 3).

A mixed ANOVA with age and gender as covariate showed no significant interaction for the three groups (F(2,84) = .26; p = .77) as well as no significant main effect of group on respiration rate (F(2,84) = .06; p = .93) during the chronic phase. In addition, there was no significant main effect of time on respiration rate (F(1,84) = .07; p = .78).

### Practice dose-response and chronic cardiovascular effects

**Fig 4:**
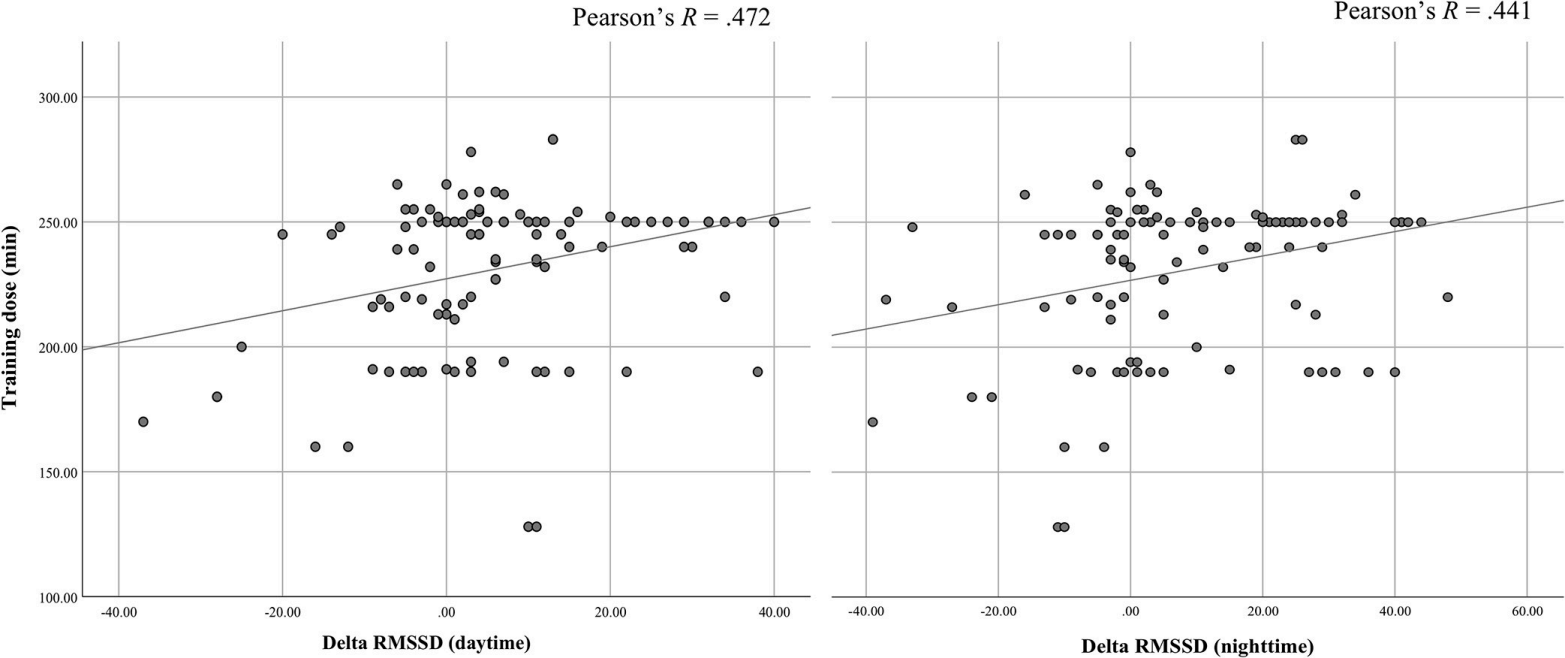
Correlation between home practice and the delta function of the chronic RMSSD phase. Correlation coefficients for the mindfulness group between total minutes of home practice and delta RMSSD during daytime (left panel) *R =* .47 and nighttime (right panel) *R* = .44. Each datapoint represents a participant.

Finally, we sought to investigate the relationship between day- and night-time RMSSD and dose-response for the music and mindfulness group (Table 1 and Fig 4). A delta variable was computed to probe whether the difference in RMSSD correlated with minutes of either mindfulness practice or music-listening. The delta variable was calculated as post RMSSD (night or day)–pre RMSSD (night or day). The Pearson correlation coefficient (*R*) for the mindfulness group’s RMSSD daytime and dose-response was significant at *R* = .47; *p* = .001; two-tailed (Fig 4, left panel), and the RMSSD during sleep and dose-response was significant at *R* = .44; *p* = .002; two-tailed (Fig 4, right panel). The results suggest that quantity of home practice had a significant impact on the change in RMSSD during day and night for the mindfulness group. For the music group there were no significant correlation for the daytime RMSSD and home practice. However, the Pearson correlation coefficient (*R*) for the music group’s RMSSD during sleep and home practice was significant at *R* = .36; *p* = .005; two-tailed (figure not shown).

## Discussion

This study has examined the impact of mindfulness practice on *chronic* as well as *acute* HRV effects compared to an active-control group and a non-intervention control group. We tested the effects of mindfulness in a *naturalistic* as opposed to a *lab-based* setting by designing a study which engaged participants in long-term HRV recordings. The study tested four hypotheses: H1) Mindfulness practice would increase the HRV response in the chronic phase during daytime in the mindfulness group post-training compared to pre-training. H2) The HRV response would increase during the acute phase over the 10-day practice period in the mindfulness group. H3) Mindfulness practice would decrease respiration rate in the acute practice phase and not in the chronic phase. H4) Mindfulness practice would increase the HRV response during sleep in the mindfulness group post-training compared to pre-training. We did find statistical support for H1, namely that the HRV would increase during the daytime in the mindfulness group. As predicted the study found support for H2, whereby the mindfulness group and surprisingly also the music group showed elevated HRV responses during the daily guided training sessions. Furthermore, we found support for H3, as we showed that respiration rate during the acute phase was reduced in the mindfulness group, but not in the chronic phase. In support of H4, we found that the mindfulness group displayed an elevated response in the HRV signal from pre- to post-intervention compared to the two other groups during sleep.

In the following we will discuss the results arising from the main hypotheses, and in addition also address the results obtained from the questionnaire data across the three groups as well as the results obtained from the training dose-response across the two active intervention groups.

### Mindfulness and respiration rate

Recent evidence has found an association between formal mindfulness practice and decreased respiration rate [33, 34, 71, 72]. Decreased respiration rate during mindfulness has also been shown to positively correlate in long-term mindfulness practitioners [73, 74]. It seems that decreased respiration rate is a general trait encountered across the mindfulness spectrum from novices to experienced practitioners. Hence, it is indeed in line with previous studies that we found attenuated respiration rate in the mindfulness group during the daily mindfulness sessions (Fig 3). We also found that the music group did not exhibit differences in respiration rate whilst listening to music, suggesting that reduced respiration rate is indeed specific to mindfulness practice. Finally, by tracking HRV both pre and post mindfulness practice, that is in periods when participants were instructed not to perform mindfulness practice, we did however not observe significant changes in respiration outside of formal mindfulness practice. Taken together, these observations suggest that attenuated respiration is specifically present during formal mindfulness practice sessions. This finding of course is in line with the concept of mindfulness where attention to breathing serves as a fundamental component of mindfulness practice [32] which corroborates the findings mentioned above from previous research demonstrating decreased respiration rate during mindfulness [33, 34, 71, 72]. In support of the abovementioned changes in respiration in the mindfulness group, we found that the acute HRV response over the course of the formal mindfulness practice sessions exhibited an increase relative a non-mindfulness baseline (Fig 2). This observation has also been reported in previous studies [21, 22, 24, 29]. However, as we found the HRV response to be elevated both in the daytime and in the nighttime in the chronic phase (Fig 1), it indicates that respiration is not solely responsible for the increases in HRV during mindfulness practice in the acute phase. That is, in the chronic phase there were no differences across the three groups (pre and post) in respiration (Fig 3), and yet the RMSSD was elevated in the mindfulness group in the chronic phases, which we discuss below.

### Mindfulness and trait-dependent effects

Mindfulness practice thus appear to be driving the changes reflected in the HRV response [31]. This physiologically mediated effect may be interpreted to reflect that the practice of mindfulness involves intentionally directing attention to one’s experience in the present moment [44], and that this repeated practice carries trait-dependent effects in the practioner in terms of being better able to center awareness on present moment experiences also in periods when no formal mindfulness practice is taking place. In support hereof, research has shown that mindfulness practice entails increase in a variety of psychological factors such as working memory, self-control, emotion regulation and attention [15, 75]. Specifically, parasympathetic influences on HRV is related to elevated cognitive control in the context of cognitive tasks [76, 77]. Mindfulness practice entails frequently becoming distracted by repetitive lapses in attention and returning the attention to the present moment, by centering awareness on the present moment experiences [45]. Presumably, over the course of training cognitive capacity gradually improves sustained attention from this repetitive exercise of focusing the attention to the present moment [36, 37]. The implication of this line of research in the context of the present study might be that increased cognitive capacity arising from mindfulness practice result in reduced susceptibility to stress during the daytime. Such an interpretation is in agreement with the observed results in the present study where increases in the HRV response during the chronic phases (day- and nighttime) were specific to the mindfulness group and not the active-control group (Fig 1). However, future studies are needed to corroborate this interpretation. For example, future studies should inspect if the elevated HRV response observed in the chronic post-intervention phase in the mindfulness is significantly higher in long-term practitioners relative to novice practitioners, and if the elevated HRV response in the chronic post-intervention phase translate into (correlate with) improved cognitive capacity.

We did not observe differences in HF-HRV and LF-HF ratios across groups during the chronic and acute phases (Table 2). These results are surprising in that previous mindfulness studies have reported differences in the frequency domain as a function of mindfulness practice [18, 19, 24, 25]. We speculate that as RMSSD has been reported to be less affected by respiratory rate as compared to frequency-domain measures [78] that this might account for the differences in the significant differences in the temporal-domain, but not in the frequency-domain in the present study. However as the previous studies mentioned above [18, 19, 24, 25] did not report both temporal- and frequency-domain measures, more studies are needed that report the whole spectrum of HRV parameters to provide a more comprehensive vision of ANS adaptation to mindfulness practice.

**Table 2:** *Chronic* and *acute* frequency-domain HRV data for the three groups, shown as mean and standard deviation collected pre and post-intervention.

	Mindfulness group	Music group	Control group
Chronic HRV variables:			
Daytime:			
HF *baseline*	3.4 ± 2.5	2.8 ± 2.5	2.7 ± 2.9
HF *post*	4.3 ± 2.8	3.2 ± 2.1	3.5 ± 2.7
LF/HF ratio *baseline*	3.2 ± 2.6	3.3 ± 2.6	2.4 ± 2.7
LF/HF ratio *post*	2.6 ± 2.4	2.6 ± 2.2	2.9 ± 3.2
Nighttime:			
HF *baseline*	4.2 ± 1.8	3.8 ± 2.1	3.9. ± 2.4
HF *post*	4.9 ± 2.1	4.3 ± 2.4	4.5. ± 2.5
LF/HF ratio *baseline*	2.8. ± 1.8	2.7. ± 2.2	2.8. ± 2.3
LF/HF ratio *post*	2.2 ± 1.9	2.5. ± 2.1.	2.6 ± 2.2
Acute HRV variables:			
HF-HRV*	4.4 ± 1.9	3.8 ± 2.5	-
LF/HF ratio*	1.4 ± 1.3	2.6 ± 3.0	-

* Mean across the 10-day intervention.

Acute data was not collected for the control group.

### Mindfulness and self-reported stress versus HRV detection of stress

We found evidence that self-reported stress was decreased over the 10-day intervention only in the mindfulness group as measured on the PSS (Table 1). This result was supported by previous findings showing reduced self-reported stress from mindfulness practice (e.g. [1–3]). Specifically, the Headspace app used in the current study have been applied in previous research demonstrating effects pertaining to stress-relief such as overt self-reported stress [56]. Our results showing a reduction on the PSS post intervention is in line with a previous study that found reduced self-reported stress on the PSS [57]. In addition, self-reported mindfulness using the MAAS has in previous research [36] been shown to be increased in line with the current results. However, the Headspace app have not previously been applied to measure covert physiological impact of stress. Importantly, as HRV has been shown to be an indicator of objective physiological stress [13–15], we hypothesized that online-based mindfulness practice on the physiological level would reflect a decreased stress response. We found that physiological stress in the daytime post-intervention was decreased (indexed as an increased HRV RMSSD response), only in the mindfulness group (Fig 1). This result indicates that participants in the mindfulness group experienced decreased objective physiological and self-perceived stress. Indeed, this result was further corroborated by a strong correlation between the time spend on daily mindfulness practice and the RMSSD (day and night) (Fig 4). Finally, self-reported mindfulness traits as measured on the MAAS was significantly elevated in the mindfulness relative to the control groups, which is supported by previous findings [53, 79]. The present study demonstrates proof-of-concept of applying real-time measurement such as HRV, which provides a fine-tuned objective assessment of a person’s state of mind and body at any given moment (even during sleep). The capability of visualizing the effects of HRV demonstrate not only that mindfulness practice exerts profound effects on the HRV response, but also how and when mindfulness exerts an impact on the underlying HRV.

### Mindfulness and sleep quality

We found an elevated HRV response during sleep in the mindfulness group relative to the two other groups. This finding extends previous findings in important ways. Specifically, previous studies have demonstrated that mindfulness practice exerts positive effects on self-perceived sleep quality [4–8]. However, no studies have to our knowledge shown that the HRV response is increased during sleep in the context of a brief 10-day mindfulness practice intervention. There were no effects on sleep quality as measured through HRV in the two other groups. Previous studies have associated poor sleep quality with elevated sympathetic activity and suppressed parasympathetic activity [50, 51]. We observed the opposite pattern in the current study namely that mindfulness practice entailed an increased HRV and thus increased physiological indicies of sleep quality. This finding was further corroborated with results from the self-report questionnarie (D3SQI) indexing sleep quality, as well as previous research [4–8], where the mindfulness group reported better sleep quality over the 10-day intervention. The mindfulness group reported significanly higer levels of sleep quality compared to the two control groups. It is however interesting that although we did not find physiological evidence of increased sleep quality in the two control groups both groups reported significantly increased sleep quality from pre to post on the D3SQI questionnaire.

Further support of the increased sleep quality reported by the mindfulness group comes from the positive correlation between the HRV response during both day-and nighttime (Fig 4). In addition, we also found that music-listening (i.e. the active control group) exhibited a positive correlation (figure not shown) with the HRV response. As poor sleep is associated with increased risk of cardiovascular disease [48] and associated with mood and anxiety symptomatology [49], it is important to investigate the salutary effects of both mindfulness and music listening as interventions aiming to increase sleep quality.

Another possibility is that mindfulness practice indeed affected attention as shown in previous research [36–39, 80] which may have reduced fatigue and thus improved sleep quality. Admittedly, this interpretation is speculative and future studies should be designed to address this possibility.

### Music and HRV

In the music group there were no significant effects observed arising from music-listening when comparing the pre HRV response to the post-intervention HRV response during the chronic phase (Fig 1). There was, however, a significant effect on the group’s acute RMSSD during the daily music sessions (Fig 2). This finding is of particular interest in that it suggests that music may elevate the physiological response, albeit to a lower degree than mindfulness. It has to our knowledge not previously been shown that music in an ecological setting, i.e. whilst participants are engaged in music listening in their home or at work over a 10-day period, can influence the HRV response. We have in our previous work demonstrated that music (specifically binaural beats) exerts positive influence over cognitive processes, albeit tested in a ‘non-ecological’ setting, i.e. in a lab-based context [80, 81]. Previous findings have reported mixed results of music’s effect on HRV [82, 83]. But studies have found acute effects of music on physiological activity indicating that the music’s frequency can affect heart rate, with some studies showing that low frequency music decreases sympathetic activity [84–88].

## Limitations

Limitations in the study included that although the non-intervention control group was requested to maintain their daily and nightly routines, we did not (as was the case in the two active intervention groups) track the non-intervention control group through daily practice cycles. Thus, we did not have probes on their activity level across the 10-day period to the same extend as in the two active intervention groups including daily-HRV acute measurements. As a potential implication, it may be that the HRV results have been skewed to reflect an elevated activity level which may have reduced their HRV response over the course of the 10-day period.

However, we did track proxies for activity levels (specifically V02 and step counts) during the chronic measurement phases which showed no significant differences across groups (S3 Table). These results suggest that differences in activity levels may not necessarily be attributed to the differences in the HRV results.

Studies have shown that respiratory rate and tidal volume exert influences in heart rate [89]. However, as we did not adjust for respiration, this could be a limitation in the study. While controlling or adjusting for respiratory influences either statistically or through breathing exercises on a theoretical level makes sense [89], it is not necessarily straightforward on a methodological level [90]. Specifically, in the current study we did not infer the role of respiratory rate which should be counted as a limitation in interpreting the results in these results.

## Conclusion

The overall goal of this study was to probe the distinction between *acute* and *chronic* cardiovascular changes in mindfulness practice. Another goal was to investigate cardiovascular effects of mindfulness in a *naturalistic* setting as opposed to a *lab-based* environment. The effects of mindfulness on cardiovascular changes were consistent with our expectations in that the results showed pronounced effects on the HRV RMSSD response during daytime and during sleep in periods when no formal mindfulness practice was taking place. Furthermore, during the daily mindfulness sessions HRV was elevated in the mindfulness group and music group. These results demonstrate causal effects of mindfulness training and provides support for the argument that a brief 10-day online-based mindfulness intervention exert positive impact on both chronic and acute effects on HRV. Finally, the work highlights the potential of applying HRV in *naturalistic* settings as a means for tracking stress regulation throughout the day.

## Supporting information

S1 Table *Chronic* HRV variables in the time and frequency domain. Data is summarized for the three groups shown as mean and standard deviation.
(DOCX)

S2 Table *Acute* HRV variables in the time and frequency domain. Data is summarized for the three groups shown as mean and standard deviation.
(DOCX)

S3 Table V02 and step count during the *chronic* phase to account for activity levels across groups. (DOCX)

S1 File Lists the demographics, physiological and questionnaire data included in the analysis. (XLSX)

S2 File Lists the physiological data included in the analysis of the *acute phase*. (XLSX)

## References

[1] Arredondo M, Sabaté M, Valveny N, Langa M, Dosantos R, Moreno J, et al A mindfulness training program based on brief practices (M-PBI) to reduce stress in the workplace: a randomised controlled pilot study. Int J Occup Environ Health. 2017;23(1):40–51. 10.1080/10773525.2017.1386607 29082831 PMC6060839

[2] Eriksson T, Germundsjö L, Åström E, Rönnlund M. Mindful Self-Compassion Training Reduces Stress and Burnout Symptoms Among Practicing Psychologists: A Randomized Controlled Trial of a Brief Web-Based Intervention. Front Psychol. 2018;9:2340 10.3389/fpsyg.2018.02340 30538656 PMC6277494

[3] Suyi Y, Meredith P, Khan A. Effectiveness of Mindfulness Intervention in Reducing Stress and Burnout for Mental Health Professionals in Singapore. Explore: The Journal of Science and Healing. 2017;13(5):319–26. 10.1016/j.explore.2017.06.001 28780213

[4] Black DS, O'Reilly GA, Olmstead R, Breen EC, Irwin MR. Mindfulness meditation and improvement in sleep quality and daytime impairment among older adults with sleep disturbances: a randomized clinical trial. JAMA Intern Med. 2015;175(4):494–501. 10.1001/jamainternmed.2014.8081 25686304 PMC4407465

[5] Garland SN, Carlson LE, Stephens AJ, Antle MC, Samuels C, Campbell TS. Mindfulness-based stress reduction compared with cognitive behavioral therapy for the treatment of insomnia comorbid with cancer: a randomized, partially blinded, noninferiority trial. J Clin Oncol. 2014;32(5):449–57. 10.1200/JCO.2012.47.7265 24395850

[6] Gross CR, Kreitzer MJ, Reilly-Spong M, Wall M, Winbush NY, Patterson R, et al Mindfulness-based stress reduction versus pharmacotherapy for chronic primary insomnia: a randomized controlled clinical trial. Explore: The Journal of Science and Healing. 2011;7(2):76–87. 10.1016/j.explore.2010.12.003 PMC3077056 21397868

[7] Hubbling A, Reilly-Spong M, Kreitzer MJ, Gross CR. How mindfulness changed my sleep: Focus groups with chronic insomnia patients. BMC Complement Altern Med. 2014;14(1):50 10.1186/1472-6882-14-50 24512477 PMC3927626

[8] Ong JC, Shapiro SL, Manber R. Combining mindfulness meditation with cognitive-behavior therapy for insomnia: A treatment-development study. Behav Ther. 2008;39(2):171–82. 10.1016/j.beth.2007.07.002 18502250 PMC3052789

[9] Van Damm NT, van Vugt MK, Vago DR, Schmalzl L, Saron CD, Olendzki A, et al Mind the Hype: A Critical Evaluation and Prescriptive Agenda for Research on Mindfulness and Meditation. Perspect Psychol Sci. 2018;13(1):36–61. 10.1177/1745691617709589 29016274 PMC5758421

[10] Baer R. Assessment of mindfulness by self-report. Curr Opin Psychol. 2019;28:42–8. 10.1016/j.copsyc.2018.10.015 30423507

[11] Baumeister RF, Vohs KD, Funder DC. Psychology as the Science of Self-Reports and Finger Movements: Whatever Happened to Actual Behavior? Perspect Psychol Sci. 2007;2(4):396–403. 10.1111/j.1745-6916.2007.00051.x 26151975

[12] Park T, Reilly-Spong M, Gross CR. Mindfulness: a systematic review of instruments to measure an emergent patient-reported outcome (PRO). Qual Life Res. 2013;22(10):2639–59. 10.1007/s11136-013-0395-8 23539467 PMC3745812

[13] Bernston GG, Bigger JT, Eckberg DL, Grossman P, Kaufmann PG, Malik M, et al Heart rate variability: origins, methods, and interpretive caveats. Psychophysiology. 1997;34:623–48. 10.1111/j.1469-8986.1997.tb02140.x 9401419

[14] Shaffer F, McCraty R, Zerr CL. A healthy heart is not a metronome: an integrative review of the heart's anatomy and heart rate variability. Front Psychol. 2014;5:1040 10.3389/fpsyg.2014.01040 25324790 PMC4179748

[15] Thayer JF, Lane RD. Claude Bernard and the heart-brain connection: further elaboration of a model of neurovisceral integration. Neurosci Biobehav Rev. 2009;33(2):81–8. 10.1016/j.neubiorev.2008.08.004 18771686

[16] Olex S, Newberg A, Figueredo VM. Meditation: should a cardiologist care? Int J Cardiol. 2013;168(3):1805–10. 10.1016/j.ijcard.2013.06.086 23890919

[17] Christodoulou G, Salami N, Black DS. The Utility of Heart Rate Variability in Mindfulness Research. Mindfulness. 2020;11(3):554–70. 10.1007/s12671-019-01296-3

[18] Adler-Neal AL, Waugh CE, Garland EL, Shaltout HA, Diz DI, Zeidan F. The Role of Heart Rate Variability in Mindfulness-Based Pain Relief. J Pain. 2019 10.1016/j.jpain.2019.07.003 31377215 PMC6994350

[19] Azam MA, Katz J, Mohabir V, Ritvo P. Individuals with tension and migraine headaches exhibit increased heart rate variability during post-stress mindfulness meditation practice but a decrease during a post-stress control condition–A randomized, controlled experiment. Int J Psychophysiol. 2016;110:66–74. 10.1016/j.ijpsycho.2016.10.011 27769879

[20] Crosswell AD, Moreno PI, Raposa EB, Motivala SJ, Stanton AL, Ganz PA, et al Effects of mindfulness training on emotional and physiologic recovery from induced negative affect. Psychoneuroendocrinology. 2017;86:78–86. 10.1016/j.psyneuen.2017.08.003 28923751 PMC5854159

[21] Delgado-Pastor LC, Perakakis P, Subramanya P, Telles S, Vila J. Mindfulness (vipassana) meditation: Effects on P3b event-related potential and heart rate variability. International Journal of Psychophysiology. 2013;90(2):207–14. 10.1016/j.ijpsycho.2013.07.006 23892096

[22] Ditto B, Eclache M, Goldman N. Short-term autonomic and cardiovascular effects of mindfulness body scan meditation. Ann Behav Med. 2006;32(3):227–34. 10.1207/s15324796abm3203_9 17107296

[23] Garland EL, Froeliger B, Howard MO. Effects of mindfulness-oriented recovery enhancement on reward responsiveness and opioid cue-reactivity. Psychopharmacology. 2014;231(16):3229–38. 10.1007/s00213-014-3504-7 24595503 PMC4111972

[24] Krygier JR, Heathers JA, Shahrestani S, Abbott M, Gross JJ, Kemp AH. Mindfulness meditation, well-being, and heart rate variability: A preliminary investigation into the impact of intensive Vipassana meditation. Int J Psychophysiol. 2013;89(3):305–13. 10.1016/j.ijpsycho.2013.06.017 23797150

[25] Lumma AL, Kok BE, Singer T. Is meditation always relaxing? Investigating heart rate, heart rate variability, experienced effort and likeability during training of three types of meditation. Int J Psychophysiol. 2015;97(1):38–45. 10.1016/j.ijpsycho.2015.04.017 25937346

[26] Murata T, Takahashi T, Hamada T, Omori M, Kosaka H, Yoshida H, et al Individual trait anxiety levels characterizing the properties of Zen meditation. Neuropsychobiology. 2004;50(2):189–94. 10.1159/000079113 15292676

[27] Peressutti C, Martin-Gonzalez JM, Garcia-Manso JM. Does mindfulness meditation shift the cardiac autonomic nervous system to a highly orderly operational state? International Journal of Cardiology. 2012;154(2):210–2. 10.1016/j.ijcard.2011.10.054 22075417

[28] Peressutti C, Martín-González JMM, García-Manso J, Mesa D. Heart rate dynamics in different levels of zen meditation. International Journal of Cardiology. 2010;145(1):142–6. 10.1016/j.ijcard.2009.06.058 19631997

[29] Tang YY, Ma Y, Fan Y, Feng H, Wang J, Feng S, et al Central and autonomic nervous system interaction is altered by short-term meditation. PNAS. 2009;106(22):8865–70. 10.1073/pnas.0904031106 19451642 PMC2690030

[30] Grossman P, Taylor EW. Toward understanding respiratory sinus arrhythmia: relations to cardiac vagal tone, evolution and biobehavioral functions. Biol Psychol. 2007;74(2):236–85. 10.1016/j.biopsycho.2005.11.014 17081672

[31] Malik M, Bigger J. T Jr, Camm AJ, Breithardt G, Cerutti S, Cohen RJ, et al Heart rate variability. standards of measurement, physiological interpretation, and clinical use. European Heart Journal. 1996;17(3):354–81. 8737210

[32] Kabat-Zinn J. Mindfulness-Based Interventions in Context: Past, Present, and Future. Clin Psychol Sci Pract. 2003;10(2):144–56.

[33] Wahbeh H, Goodrich E, Goy E, Oken BS. Mechanistic Pathways of Mindfulness Meditation in Combat Veterans With Posttraumatic Stress Disorder: Mechanistic Pathways of Mindfulness Meditation. Journal of clinical psychology. 2016;72(4):365–83. 10.1002/jclp.22255 26797725 PMC4803530

[34] Ahani A, Wahbeh H, Nezamfar H, Miller M, Erdogmus D, Oken B. Quantitative change of EEG and respiration signals during mindfulness meditation. Journal of neuroengineering and rehabilitation. 2014;11(1):87 10.1186/1743-0003-11-87 24939519 PMC4060143

[35] Farb NAS, Segal ZV, Mayberg H, Bean J, McKeon D, Fatima Z, et al Attending to the present: mindfulness meditation reveals distinct neural modes of self-reference. Social cognitive and affective neuroscience. 2007;2(4):313–22. 10.1093/scan/nsm030 18985137 PMC2566754

[36] Bennike IH, Wieghorst A, Kirk U. Online-based mindfulness training reduces mind wandering. J Cognitive Enhancement. 2017;1(2):172–81.

[37] Jha AP, Morrison AB, Dainer-Best J, Parker S, Rostrup N, Stanley EA. Minds "at attention": mindfulness training curbs attentional lapses in military cohorts. PLoS One. 2015;10(2). 10.1371/journal.pone.0116889 PMC4324839 25671579

[38] Mrazek MD, Franklin MS, Phillips DT, Baird B, Schooler JW. Mindfulness training improves working memory capacity and GRE performance while reducing mind wandering. Psychological Science. 2013;24:776–81. 10.1177/0956797612459659 23538911

[39] Zeidan F, Johnson SK, Diamond BJ, David Z, Goolkasian P. Mindfulness meditation improves cognition: evidence of brief mental training. Conscious Cogn. 2010;19(2):597–605. 10.1016/j.concog.2010.03.014 20363650

[40] Lutz A, Slagter HA, Dunne JD, Davidson RJ. Attention regulation and monitoring in meditation. Trends in cognitive sciences. 2008;12(4):163–9. 10.1016/j.tics.2008.01.005 18329323 PMC2693206

[41] Kirk U, Gu X, Harvey AH, Fonagy P, Montague PR. Mindfulness training modulates value signals in ventromedial prefrontal cortex through input from insular cortex. NeuroImage (Orlando, Fla). 2014;100:254–62. 10.1016/j.neuroimage.2014.06.035 24956066 PMC4140407

[42] Kirk U, Pagnoni G, Hétu S, Montague R. Short-term mindfulness practice attenuates reward prediction errors signals in the brain. Scientific reports. 2019;9(1):6964–8. 10.1038/s41598-019-43474-2 31061515 PMC6502850

[43] Thayer JF, Ahs F, Fredrikson M, Sollers J Jr, Wager TD. A meta-analysis of heart rate variability and neuroimaging studies: implications for heart rate variability as a marker of stress and health-. Neurosci Biobehav Rev. 2012;36(2):747–56. 10.1016/j.neubiorev.2011.11.009 22178086

[44] Bishop SR, Lau M, Shapiro S, Carlson L, Anderson ND, Carmody J, et al Mindfulness: A proposed operational definition. Clinical Psychology: Science and Practice. 2004;11(3):230–41.

[45] Puddicombe A. Get Some Headspace: 10 Minutes Can Make All The Difference. London: Hodder & Stoughton; 2011 216 p.

[46] Jerath R, Edry JW, Barnes VA, Jerath V. Physiology of long pranayamic breathing: Neural respiratory elements may provide a mechanism that explains how slow deep breathing shifts the autonomic nervous system. Medical hypotheses. 2006;67(3):566–71. 10.1016/j.mehy.2006.02.042 16624497

[47] Schmidt MH. The energy allocation function of sleep: A unifying theory of sleep, torpor, and continuous wakefulness. Neuroscience & Biobehavioral Reviews. 2014;47:122–53. 25117535 10.1016/j.neubiorev.2014.08.001

[48] Mallon L, Broman JE, Hetta J. Sleep complaints predict coronary artery disease mortality in males: a 12‐year follow‐up study of a middle‐aged Swedish population. Journal of Internal Medicine. 2002;251(3):207–16. 10.1046/j.1365-2796.2002.00941.x 11886479

[49] Wipawan Chaoum P, Somrat L, Vitool L, Thanapoom R, Thanawan S, Gelaye B, et al Relationship between Poor Sleep Quality and Psychological Problems among Undergraduate Students in the Southern Thailand. Walailak Journal of Science & Technology. 2016;13(4):235–42. 27152114 PMC4853815

[50] Barone DA, Krieger AC. The Function of Sleep, review. AIMS Neuroscience. 2015;2(2):71–90.

[51] Kesek M., Franklin K. A., Sahlin C, Lindberg E. Heart rate variability during sleep and sleep apnoea in a population based study of 387 women. Clin Physiol Funct Imaging. 2009;29(4):309–15. 19453563 10.1111/j.1475-097X.2009.00873.x

[52] Cohen S, Kamarck T, Mermelstein R. A global measure of perceived stress. Journal of Health and Social Behavior. 1983;24(4):386–96. 6668417

[53] Brown KW, Ryan RM. The benefits of being present: Mindfulness and its role in psychological well-being. Journal of Personality and Social Psychology. 2003;84(4):822–48. 10.1037/0022-3514.84.4.822 12703651

[54] Ralevski E, Petrakis I, Altemus M. Heart rate variability in alcohol use: A review. Pharmacol Biochem Behav. 2019;176:83–92. 10.1016/j.pbb.2018.12.003 30529588

[55] Sjoberg N, Saint DA. A single 4 mg dose of nicotine decreases heart rate variability in healthy nonsmokers: implications for smoking cessation programs. Nicotine & Tobacco Research. 2011;13(5):369–72. 10.1093/ntr/ntr004 21350044

[56] Economides M, Martman J, Bell MJ, Sanderson B. Improvements in Stress, Affect, and Irritability Following Brief Use of a Mindfulness-based Smartphone App: A Randomized Controlled Trial. Mindfulness. 2018;9(5):1584–93. 10.1007/s12671-018-0905-4 30294390 PMC6153897

[57] Champion L, Economides M, Chandler C. The efficacy of a brief app-based mindfulness intervention on psychosocial outcomes in healthy adults: A pilot randomised controlled trial. PloS one. 2018;13(12):e0209482 10.1371/journal.pone.0209482 30596696 PMC6312207

[58] Howells A, Ivtzan I, Eiroa-Orosa FJ. Putting the ‘app’ in Happiness: A Randomised Controlled Trial of a Smartphone-Based Mindfulness Intervention to Enhance Wellbeing. Journal of happiness studies. 2014;17(1):163–85. 10.1007/s10902-014-9589-1

[59] Wylde CM, Mahrer NE, Meyer RML, Gold JI. Mindfulness for Novice Pediatric Nurses: Smartphone Application Versus Traditional Intervention. Journal of pediatric nursing. 2017;36:205–12. 10.1016/j.pedn.2017.06.008 28888505

[60] Wen L, Sweeney TE, Welton L, Trockel M, Katznelson L. Encouraging Mindfulness in Medical House Staff via Smartphone App: A Pilot Study. Academic psychiatry. 2017;41(5):646–50. 10.1007/s40596-017-0768-3 28795335

[61] Tang Y-Y, Ma Y, Wang J, Fan Y, Feng S, Lu Q, et al Short-term meditation training improves attention and self-regulation. Proceedings of the National Academy of Sciences—PNAS. 2007;104(43):17152–6. 10.1073/pnas.0707678104 17940025 PMC2040428

[62] Kirk U, Gu X, Sharp C, Hula A, Fonagy P, Montague PR. Mindfulness training increases cooperative decision making in economic exchanges: Evidence from fMRI. NeuroImage (Orlando, Fla). 2016;138:274–83. 10.1016/j.neuroimage.2016.05.075 27266443 PMC4954868

[63] Spiesshoefer J, Linz D, Skobel E, Arzt M, Stadler S, Schoebel C, et al Sleep–the yet underappreciated player in cardiovascular diseases: A clinical review from the German Cardiac Society Working Group on Sleep Disordered Breathing. European journal of preventive cardiology. 2019:204748731987952 10.1177/2047487319879526 33611525

[64] Buysse DJ, Reynolds CF, Monk TH, Berman SR, Kupfer DJ. The Pittsburgh Sleep Quality Index: a new instrument for psychiatric practice and research. Psychiatry Research. 1989;28(2):193–213. 10.1016/0165-1781(89)90047-4 2748771

[65] Parak J, Korhonen I. Accuracy of Firstbeat Bodyguard 2 beat-to-beat heart rate monitor, (whitepaper). 2013.

[66] Parak J, Tarniceriu A, Renevey P, Bertschi M, Delgado-Gonzalo R, Korhonen I. Evaluation of the beat-to-beat detection accuracy of PulseOn wearable optical heart rate monitor. Conf Proc IEEE Eng Med Biol Soc 2015 p. 8099–102. 10.1109/EMBC.2015.7320273 26738173

[67] Ottaviani C, Shahabi L, Tarvainen M, Cook I, Abrams M, Shapiro D. Cognitive, behavioral, and autonomic correlates of mind wandering and perseverative cognition in major depression. Frontiers in neuroscience. 2015;8:433 10.3389/fnins.2014.00433 25601824 PMC4283544

[68] Tarvainen MP, Niskanen J-P, Lipponen JA, Ranta-aho PO, Karjalainen PA. Kubios HRV–Heart rate variability analysis software. Computer methods and programs in biomedicine. 2014;113(1):210–20. 10.1016/j.cmpb.2013.07.024 24054542

[69] Cohen J. Statistical Power Analysis for the Behavioral Sciences. 2nd ed Abingdon: Routledge; 1988 400 p.

[70] Bakeman R. Recommended effect size statistics for repeated measures designs. Behavior Research Methods. 2005;37(3):379–84. 10.3758/bf03192707 16405133

[71] Farb NAS, Segal ZV, Anderson AK. Mindfulness meditation training alters cortical representations of interoceptive attention. Soc Cogn Affect Neurosci. 2013;8(1):15–26. 10.1093/scan/nss066 22689216 PMC3541492

[72] Zeidan F, Emerson NM, Farris SR, Ray JN, Jung Y, McHaffie JG, et al Mindfulness meditation-based pain relief employs different neural mechanisms than placebo and sham mindfulness meditation-induced analgesia. J Neurosci. 2015;35:15307–25. 10.1523/JNEUROSCI.2542-15.2015 26586819 PMC4649004

[73] Lazar SW, Kerr CE, Wasserman RH, Gray JR, Greve DN, Treadway MT, et al Meditation experience is associated with increased cortical thickness. Neuroreport. 2005;16(17):1893–7. 10.1097/01.wnr.0000186598.66243.19 16272874 PMC1361002

[74] Wielgosz J, Schuyler BS, Lutz A, Davidson RJ. Long-term mindfulness training is associated with reliable differences in resting respiration rate. Sci Rep. 2016;6(1):27533 10.1038/srep27533 27272738 PMC4895172

[75] Segerstrom SC, Nes LS. Heart rate variability reflects self-regulatory strength, effort, and fatigue. Psychol Sci. 2007;18(3):275–81. 10.1111/j.1467-9280.2007.01888.x 17444926

[76] Hansen AL, Johnsen BH, Thayer JF. Vagal influence on working memory and attention. Int J Psychophysiol. 2003;48(3):263–74. 10.1016/s0167-8760(03)00073-4 12798986

[77] Overbeek TJ, van Boxtel A, Westerink JH. Respiratory sinus arrhythmia responses to cognitive tasks: effects of task factors and RSA indices. Biol Psychol. 2014;99(1):1–14. 10.1016/j.biopsycho.2014.02.006 24561100

[78] Hill LK, Siebenbrock A. All are measures created equal? Heart rate variability and respiration. Biomed Sci Instrum. 2009;45:71–6. 19369742

[79] Sun S, Hu C, Pan J, Liu C, Huang M. Trait Mindfulness Is Associated With the Self-Similarity of Heart Rate Variability. Frontiers in psychology. 2019;10:314 10.3389/fpsyg.2019.00314 30873070 PMC6403186

[80] Axelsen JL, Staiano W, Kirk U. On-the-spot binaural beats and mindfulness reduces the effect of mental fatigue. J of Cogn Enhancement 2020;41(1):31–9. 10.1007/s41465-019-00162-3

[81] Kirk U, Wieghorst A, Nielsen CM, Staiano W. On-the-Spot Binaural Beats and Mindfulness Reduces Behavioral Markers of Mind Wandering. Journal of Cognitive Enhancement. 2019.

[82] Amaral JA, Guida HL, Nogueira ML, Roque AL, Abreu LC, Raimundo RD, et al Musical auditory stimulation at different intensities and its effects on the geometric indices of heart‐rate variability. Focus on Alternative and Complementary Therapies. 2014;19(3):132–9. 10.1111/fct.12124

[83] McConnell PA, Froeliger B, Garland EL, Ives JC, Sforzo GA. Auditory driving of the autonomic nervous system: Listening to theta-frequency binaural beats post-exercise increases parasympathetic activation and sympathetic withdrawal. Front Psychol. 2014;5:1248 10.3389/fpsyg.2014.01248 25452734 PMC4231835

[84] Bradt J, Dileo C, Magill L, Teague A. Music interventions for improving psychological and physical outcomes in cancer patients. Cochrane library. 2016;2016(8):CD006911 10.1002/14651858.CD006911.pub3 27524661

[85] Nakajima Y, Tanaka N, Mima T, Izumi S-I. Stress Recovery Effects of High- and Low-Frequency Amplified Music on Heart Rate Variability. Behavioural neurology. 2016;2016:5965894–8. 10.1155/2016/5965894 27660396 PMC5021883

[86] Wulff V, Hepp P, Fehm T, Schaal N. Music in Obstetrics: An Intervention Option to Reduce Tension, Pain and Stress. Geburtshilfe und Frauenheilkunde. 2017;77(9):967–75. 10.1055/s-0043-118414 28959060 PMC5612774

[87] Halbert JD, Tuyll DR, Purdy C, Hao G, Cauthron S, Crookall C, et al Low Frequency Music Slows Heart Rate and Decreases Sympathetic Activity. Music & Medicine. 2018;10(4):180–5.

[88] Ooishi Y, Mukai H, Watanabe K, Kawato S, Kashino M. Increase in salivary oxytocin and decrease in salivary cortisol after listening to relaxing slow-tempo and exciting fast-tempo music. PloS One. 2017;12(12):e0189075 10.1371/journal.pone.0189075 29211795 PMC5718605

[89] Brown TE, Beightol LA, Koh J, Eckberg DL. Important influence of respiration on human R-R interval power spectra is largely ignored. J Appl Physiol (1985). 1993 11;75(5):2310–7. 10.1152/jappl.1993.75.5.2310 . 8307890

[90] de Geus EJC, Gianaros PJ, Brindle RC, Jennings JR, Berntson GG. Should heart rate variability be "corrected" for heart rate? Biological, quantitative, and interpretive considerations. Psychophysiology. 2019 2;56(2):e13287 10.1111/psyp.13287 Epub 2018 Oct 25. ; PMCID: PMC6378407. 30357862 PMC6378407

