# Supplementary material for: Heart rate variability is enhanced during mindfulness practice: A randomized controlled trial involving a 10-day online-based mindfulness intervention
Source: PLoS One. 2020 Dec 17;15(12):e0243488. doi: 10.1371/journal.pone.0243488 (PMC7746169; doi:10.1371/journal.pone.0243488)
Supplement: S1 Table — Data is summarized for the three groups shown as mean and standard deviation. (DOCX) [file pone.0243488.s001.docx]

**S1 Table.** *Chronic* HRV variables in the time and frequency domain not included in the primary analysis. Data is summarized for the three groups during daytime shown as mean and standard deviation.

|  | **Mindfulness group** | **Music group** | **Control group** |
| --- | --- | --- | --- |
| Time domain: |  |  |  |
| SDNN *baseline* | 123.4 ± 24.3 | 114.5 ± 28.5 | 124.3 ± 32.1 |
| SDNN *post* | 134.3 ± 30.3 | 121.5 ± 23.9 | 130.4 ± 32.6 |
| SDANN *baseline* | 113.9 ± 24.1 | 108.1 ± 25.2 | 115.9 ± 26.9 |
| SDANN *post* | 115.5 ± 24.9 | 118.7 ± 29.2 | 122.6 ± 30.3 |
| pNN50 *baseline* | 19.8 ± 11.2 | 18.1 ± 9.8 | 18.3 ± 14.8 |
| pNN50 *post* | 22.3 ± 11.9 | 22.6 ± 13.1 | 18.8 ± 12.9 |
| Frequency domain: |  |  |  |
| LF *baseline* | 6.2 ± 3.6 | 5.8 ± 2.9 | 5.8 ± 3.1 |
| LF *Post*  VLF *baseline*  VLF *post* | 5.9 ± 2.8  5.5±0.8  5.4 ± 1.1 | 6.1 ± 2.9  5.9±1.1  5.8 ± 1.3 | 5.6 ± 2.9  5.8±1.3  6.0 ± 1.1 |

*There were no statistically significant differences within or across groups (p < 0.05).*
