# Supplementary material for: Heart rate variability is enhanced during mindfulness practice: A randomized controlled trial involving a 10-day online-based mindfulness intervention
Source: PLoS One. 2020 Dec 17;15(12):e0243488. doi: 10.1371/journal.pone.0243488 (PMC7746169; doi:10.1371/journal.pone.0243488)
Supplement: S2 Table — Data is summarized for the three groups shown as mean and standard deviation. (DOCX) [file pone.0243488.s002.docx]

**S2 Table.** *Acute* HRV variables in the time and frequency domain not included in the primary analysis. Data is summarized for the three groups shown as mean and standard deviation.

|  | **Mindfulness group** | **Music group** | **Control group** |
| --- | --- | --- | --- |
| Time domain: |  |  |  |
| SDNN | 133.5 ± 29.1 | 122.7 ± 25.9 | - |
| pNN50 | 23.9 ± 13.2 | 18.4 ± 14.3 | - |
| Frequency domain: |  |  |  |
| LF | 4.8 ± 1.8 | 4.1 ± 1.9 | - |
| VLF | 5.4 ± 1.0 | 5.8 ±1.2 | - |

*There were no statistically significant differences within or across groups (p < 0.05). Acute data was not collected for the control group.*
