# Supplementary material for: Heart rate variability is enhanced during mindfulness practice: A randomized controlled trial involving a 10-day online-based mindfulness intervention
Source: PLoS One. 2020 Dec 17;15(12):e0243488. doi: 10.1371/journal.pone.0243488 (PMC7746169; doi:10.1371/journal.pone.0243488)
Supplement: S3 Table — (DOCX) [file pone.0243488.s003.docx]

**S3 Table.** V02 and step count during the *chronic* phase to account for activity levels across groups. Variables are separated for day and night during baseline and post. Data is summarized for the three groups shown as mean and standard deviation.

|  | **Mindfulness group** | **Music group** | **Control group** |
| --- | --- | --- | --- |
| Daytime: |  |  |  |
| V02 *baseline* | 5.4 ± 0.7 | 5.5 ± 0.9 | 5.3 ± 0.8 |
| V02 *post* | 5.6 ± 0.8 | 5.4 ± 0.6 | 5.4 ± 0.6 |
| Steps *baseline* | 7767 ± 434 | 7965 ± 467 | 7654 ± 473 |
| Steps *post* | 7645 ± 419 | 7657 ± 432 | 7890 ± 443 |
| Nighttime: |  |  |  |
| V02 *baseline* | 3.2 ± 0.5 | 3.4 ± 0.5 | 3.4 ± 0.6 |
| V02 *post* | 3.4 ± 0.4 | 3.2 ± 0.6 | 3.5 ± 0.5 |
| Steps *baseline* | 27 ± 14 | 26 ± 16 | 25 ± 15 |
| Steps *post* | 32 ± 13 | 31 ± 14 | 27 ± 17 |

*There were no statistically significant differences within or across groups for the variables V02 (baseline and post) and Steps (baseline and post) (p < 0.05).*
